# Ferroptosis in Lymphoproliferative Disorders

**DOI:** 10.3390/cells15131184

**Published:** 2026-06-29

**Authors:** Santino Caserta, Enrica Antonia Martino, Ernesto Vigna, Antonella Bruzzese, Mamdouh Skafi, Nicola Amodio, Eugenio Lucia, Virginia Olivito, Caterina Labanca, Francesco Mendicino, Maria Eugenia Alvaro, Fortunato Morabito, Massimo Gentile

**Affiliations:** 1 Hematology Unit, Department of Onco-Hematology, Azienda Ospedaliera of Cosenza, 87100 Cosenza, Italy; 2 Emergency and Internal Medicine Department, Saint Joseph Hospital, East Jerusalem 9720000, Palestine; 3 Department of Experimental and Clinical Medicine, University of Catanzaro, 88100 Catanzaro, Italy; 4 Scientific Committee Dafne SRL, 89048 Siderno, Italy; 5 Department of Pharmacy, Health and Nutritional Science, University of Calabria, 87036 Rende, Italy

**Keywords:** ferroptosis, cancer biology, lymphoproliferative disorders, lymphoma

## Abstract

Ferroptosis is a regulated form of cell death driven by iron-dependent lipid peroxidation and is mechanistically distinct from apoptosis, necrosis and pyroptosis. Increasing evidence indicates that ferroptosis plays a critical role in cancer biology, including lymphoproliferative disorders, where chronic redox imbalance, dysregulated iron metabolism, and metabolic rewiring create a permissive environment for ferroptotic vulnerability. In these malignancies, altered iron handling, elevated reactive oxygen species, and a strong reliance on antioxidant systems such as glutathione and glutathione peroxidase 4 tightly control ferroptotic sensitivity. Dysregulation of key components, including SLC7A11, lipid metabolism pathways, and intracellular iron homeostasis, further shapes the susceptibility of malignant lymphoid cells to ferroptosis. Importantly, emerging preclinical studies suggest that therapeutic targeting of ferroptosis may overcome resistance to conventional chemotherapy, targeted agents, and immunotherapy, offering novel opportunities particularly in relapsed or refractory disease. This review provides a comprehensive overview of the molecular mechanisms governing ferroptosis in lymphoproliferative disorders, highlights the interplay between ferroptosis and major cellular and metabolic pathways, and discusses current and emerging strategies to pharmacologically induce ferroptosis, with an emphasis on biomarker-driven clinical translation.

## 1. Introduction

Regulated cell death (RCD) is a fundamental biological process that ensures tissue homeostasis and eliminates damaged or malignant cells. Over the past decades, multiple forms of RCD have been characterized, including apoptosis, necroptosis, and pyroptosis, each defined by distinct molecular mechanisms and morphological features. Among these, ferroptosis has emerged as a unique iron-dependent form of cell death driven by the excessive accumulation of lipid peroxides to lethal levels. First described in 2012, ferroptosis is mechanistically and biochemically distinct from other cell death modalities, as it is primarily governed by cellular iron metabolism, redox homeostasis, and lipid peroxidation dynamics [1].

At the molecular level, ferroptosis is triggered by an imbalance between the production of reactive oxygen species (ROS) and the cellular antioxidant defense systems. Central to this process is the glutathione (GSH)–glutathione peroxidase 4 (GPX4) axis, which detoxifies lipid hydroperoxides and prevents membrane damage. Disruption of this pathway—through inhibition of cystine uptake via system Xc^−^ (SLC7A11/SLC3A2), depletion of GSH, or direct inactivation of GPX4—leads to uncontrolled lipid peroxidation and ultimately cell death. In parallel, intracellular iron availability plays a crucial role by catalyzing Fenton reactions that generate highly reactive radicals, thereby amplifying oxidative damage. Additional regulatory pathways, including the ferroptosis suppressor protein 1 (FSP1)–coenzyme Q10 system and mitochondrial metabolic processes, further modulate ferroptotic sensitivity [2].

In cancer biology, ferroptosis has gained increasing attention as both a tumor-suppressive mechanism and a potential therapeutic vulnerability. Importantly, the relevance of ferroptosis extends far beyond hematologic malignancies. Dysregulated ferroptotic pathways have been described in numerous solid tumors, including hepatocellular carcinoma, pancreatic cancer, lung cancer, breast cancer, colorectal cancer, glioblastoma, and sarcomas. In these settings, altered iron metabolism, oxidative stress, and lipid remodeling contribute to ferroptotic susceptibility and represent promising therapeutic targets. This broad involvement highlights ferroptosis as a fundamental mechanism across cancer biology rather than a phenomenon restricted to lymphoid neoplasms [3]. Cancer cells often exhibit profound metabolic reprogramming, characterized by enhanced iron uptake, elevated ROS levels, and a heightened reliance on antioxidant systems to maintain redox balance. These features can render tumor cells particularly susceptible to ferroptotic cell death under conditions of oxidative stress. However, malignant cells also develop adaptive mechanisms to evade ferroptosis, such as upregulation of SLC7A11, activation of NRF2-dependent antioxidant responses, and extensive remodeling of lipid metabolism.

Lymphoproliferative disorders (LPDs), including lymphomas, chronic lymphocytic leukemia (CLL), and multiple myeloma (MM), represent a heterogeneous group of malignancies arising from lymphoid cells at different stages of differentiation. These diseases are characterized by complex genetic alterations and significant metabolic rewiring, often associated with increased oxidative stress and dysregulated iron homeostasis. Emerging evidence suggests that such features create a permissive environment for ferroptotic vulnerability while simultaneously promoting resistance through enhanced antioxidant capacity. Despite substantial therapeutic advances, many patients with lymphoproliferative malignancies eventually develop resistance to conventional chemotherapy, targeted agents, or immunotherapy, highlighting the need for novel treatment strategies that exploit non-apoptotic forms of regulated cell death [4].

In this context, ferroptosis has been proposed as a promising avenue to overcome therapeutic resistance and selectively target malignant lymphoid cells. Preclinical studies have demonstrated that pharmacological induction of ferroptosis can effectively suppress tumor growth, restore sensitivity to existing treatments, and synergize with immunotherapy in several models. Nevertheless, the precise role of ferroptosis in lymphoproliferative disorders remains incompletely understood, and several challenges—including the identification of reliable and disease-specific biomarkers, definition of ferroptosis-addicted subsets, and the development of safe and effective ferroptosis-inducing agents—must be addressed before broad clinical translation.

This review aims to provide a comprehensive overview of the molecular mechanisms regulating ferroptosis, with a particular focus on their relevance in lymphoproliferative disorders. We discuss the interplay between ferroptosis and key cellular pathways, examine current evidence linking ferroptosis to disease progression and therapy resistance across major lymphoid entities, and highlight emerging therapeutic strategies designed to exploit ferroptotic vulnerabilities. Particular attention is given to potential biomarkers and to the challenges and opportunities associated with incorporating ferroptosis-targeting approaches into biomarker-driven clinical trials in lymphoid malignancies.

## 2. Molecular Mechanisms of Ferroptosis

Ferroptosis is driven by a tightly regulated interplay between iron metabolism, lipid peroxidation, and antioxidant defense systems. The convergence of these processes determines the susceptibility of cells to undergoing ferroptotic death, with iron-dependent oxidative damage to membrane lipids representing the central execution mechanism.

### 2.1. Iron Metabolism and Homeostasis

Iron is essential for numerous cellular processes, including DNA synthesis, mitochondrial respiration, and redox reactions. However, excess free iron can catalyze the formation of highly reactive radicals, thereby promoting oxidative damage and ferroptosis.

Circulating ferric iron (Fe^3+^) is bound to transferrin and internalized through transferrin receptor 1 (TFRC)-mediated endocytosis. Within endosomes, Fe^3+^ is reduced to ferrous iron (Fe^2+^) and released into the cytosol via divalent metal transporter 1 (DMT1). This process contributes to the intracellular labile iron pool (LIP), which is critical for ferroptotic signaling [5].

To prevent iron-induced toxicity, excess intracellular iron is stored in ferritin, a multimeric protein complex composed of heavy (FTH1) and light (FTL) chains. Ferritin sequesters iron in a redox-inactive form, thereby limiting its participation in radical-generating reactions. Conversely, iron export is mediated by ferroportin (SLC40A1), the only known cellular iron exporter, which maintains systemic and intracellular iron balance. Dysregulation of ferritin turnover, particularly through selective autophagic degradation (ferritinophagy), can increase free iron availability and sensitize cells to ferroptosis [6].

The labile iron pool consists mainly of redox-active ferrous iron (Fe^2+^), which participates in the Fenton reaction. In this process, Fe^2+^ reacts with hydrogen peroxide (H_2_O_2_) generating ferric iron (Fe^3+^), hydroxyl radicals (•OH), and hydroxide ions. Hydroxyl radicals are highly reactive species capable of initiating lipid peroxidation chain reactions, ultimately leading to membrane damage and ferroptotic cell death. Continuous recycling of Fe^3+^ back to Fe^2+^ through intracellular reductive systems further amplifies oxidative stress and propagates ferroptosis [7].

### 2.2. Lipid Peroxidation

Lipid peroxidation is the central execution step of ferroptosis and involves the oxidative degradation of membrane phospholipids containing polyunsaturated fatty acids (PUFAs). The accumulation of lipid hydroperoxides ultimately disrupts membrane integrity and triggers cell death.

PUFAs are particularly susceptible to peroxidation due to the presence of bis-allylic hydrogen atoms. Their incorporation into membrane phospholipids is a prerequisite for ferroptosis, as saturated and monounsaturated fatty acids are less prone to oxidation. The abundance and composition of PUFA-containing phospholipids therefore critically influence ferroptotic vulnerability [5].

Key enzymes regulate the synthesis and oxidation of PUFA-containing phospholipids. Acyl-CoA synthetase long-chain family member 4 (ACSL4) facilitates the activation of PUFAs into acyl-CoA derivatives, while lysophosphatidylcholine acyltransferase 3 (LPCAT3) incorporates these lipids into membrane phospholipids. Lipoxygenases (LOXs) further catalyze the oxygenation of PUFAs, contributing to the accumulation of lipid hydroperoxides. The expression and activity of these enzymes are closely linked to ferroptotic sensitivity [7].

In addition to enzymatic processes, lipid peroxidation can occur through non-enzymatic, free radical-mediated chain reactions driven by iron-dependent ROS. Once initiated, these reactions propagate rapidly across membrane lipids, amplifying oxidative damage and reinforcing ferroptotic cell death.

### 2.3. Antioxidant Defense Systems

Cells possess multiple antioxidant systems that counteract lipid peroxidation and protect against ferroptosis. The balance between these protective mechanisms and oxidative stress determines whether ferroptosis is initiated.

Glutathione is a major intracellular antioxidant synthesized from cysteine, glutamate, and glycine. It serves as a cofactor for several detoxifying enzymes and plays a central role in maintaining redox homeostasis. The availability of cysteine, often imported in its oxidized form (cystine), is a limiting factor for GSH synthesis and thus for ferroptosis resistance.

Glutathione peroxidase 4 (GPX4) is the key enzyme that reduces lipid hydroperoxides to non-toxic lipid alcohols using GSH as a cofactor. GPX4 activity is essential for preventing ferroptosis, and its inhibition—either directly or through GSH depletion—leads to the accumulation of lipid peroxides and ferroptotic cell death [8].

System Xc^−^ is a cystine/glutamate antiporter composed of the subunits SLC7A11 and SLC3A2. It imports extracellular cystine in exchange for intracellular glutamate, thereby supporting intracellular cysteine levels and GSH synthesis. Inhibition of system Xc^−^ reduces GSH availability and sensitizes cells to ferroptosis, whereas overexpression of SLC7A11 is frequently observed in cancer and contributes to ferroptosis resistance.

In addition to the GPX4-dependent system, several parallel antioxidant pathways suppress ferroptosis. Ferroptosis suppressor protein 1 (FSP1) reduces coenzyme Q10 (CoQ10) to its active antioxidant form, which prevents lipid peroxidation in cellular membranes. Dihydroorotate dehydrogenase (DHODH) provides a similar protective function in mitochondria. Furthermore, the GTP cyclohydrolase 1 (GCH1)–tetrahydrobiopterin (BH4) axis acts as a potent radical-trapping antioxidant system, limiting lipid oxidation independently of GPX4 [9].

The dynamic interplay between iron handling, lipid metabolism, and antioxidant defenses establishes the molecular framework of ferroptosis. Dysregulation of any of these components can shift the cellular balance toward either resistance or susceptibility to ferroptotic cell death, with important implications for cancer biology and therapeutic targeting.

Molecular mechanisms of ferroptosis are explicated in Table 1.

## 3. Biological Features of Lymphoproliferative Disorders

LPDs encompass a heterogeneous group of malignancies derived from lymphoid cells at various stages of differentiation, including indolent and aggressive B-cell and T-cell lymphomas, CLL, and MM.

Malignant lymphoid cells exhibit profound metabolic reprogramming to support continuous proliferation and survival under cellular stress. Alterations in iron metabolism lead to an expanded labile iron pool that promotes ROS generation through Fenton chemistry. Concomitantly, malignant cells display enhanced glycolysis and glutaminolysis, which provide substrates for biosynthetic pathways and antioxidant defenses. Lipid metabolism is also significantly remodeled, with increased incorporation of polyunsaturated fatty acids into membrane phospholipids, a change that predisposes cells to lipid peroxidation. Additional metabolic adaptations, including activation of the mevalonate pathway and modifications of mitochondrial function, further support redox balance and membrane integrity, highlighting the interplay between cellular metabolism and susceptibility to regulated forms of cell death such as ferroptosis.

The redox status of lymphoid malignancies is typically skewed toward oxidative stress. Elevated ROS levels originate from both intrinsic mitochondrial activity, oncogenic signaling, and dysregulated iron handling. While moderate ROS concentrations can act as signaling molecules that promote proliferation and survival, excessive ROS accumulation causes oxidative damage to lipids, proteins, and nucleic acids. This redox imbalance is further exacerbated by alterations in ferritin turnover and iron export, which increase the availability of redox-active iron and enhance susceptibility to lipid peroxidation, a central mechanism of ferroptotic cell death. As a consequence, malignant lymphoid cells are under constant pressure to activate adaptive antioxidant responses [10].

To counteract the detrimental effects of ROS and prevent ferroptotic death, malignant lymphoid cells rely on multiple interconnected antioxidant systems. The glutathione–glutathione peroxidase 4 (GPX4) axis plays a central role in detoxifying lipid hydroperoxides and maintaining membrane integrity. Cellular uptake of cystine through the system Xc^−^ antiporter, composed of SLC7A11 and SLC3A2 subunits, ensures the availability of cysteine for glutathione synthesis, and upregulation of this transporter is a common feature in LPDs, conferring resistance to oxidative stress. Beyond the GPX4-dependent system, additional mechanisms, including the FSP1–coenzyme Q10 axis, dihydroorotate dehydrogenase, and the GCH1–tetrahydrobiopterin pathway, provide complementary antioxidant capacity, allowing malignant lymphoid cells to survive in conditions of high oxidative pressure [11]. Taken together, these metabolic and redox adaptations create a state of “redox addiction”, in which tumor survival critically depends on ferroptosis-suppressive pathways and thereby generates exploitable vulnerabilities.

Beyond hematologic malignancies, ferroptosis has been extensively implicated in the biology of several solid tumors, including hepatocellular carcinoma, lung cancer, pancreatic cancer, breast cancer, colorectal cancer, glioblastoma, and soft tissue sarcomas. In these malignancies, alterations in iron metabolism, lipid remodeling, and antioxidant pathways create context-dependent ferroptotic vulnerabilities that may be therapeutically exploited. Consequently, ferroptosis is increasingly recognized as a universal hallmark of cancer biology rather than a phenomenon restricted to lymphoid neoplasms [12].

## 4. Ferroptosis in Lymphoproliferative Malignancies

Increasing evidence indicates that ferroptosis plays a significant role in the biology of LPDs, where profound metabolic alterations and chronic oxidative stress create a permissive environment for ferroptotic regulation. Malignant lymphoid cells exhibit marked dependence on iron metabolism, antioxidant defenses, and lipid remodeling pathways, all of which critically influence susceptibility to ferroptotic cell death. Although these adaptations support tumor growth and survival, they also generate specific vulnerabilities that may be therapeutically exploited. In recent years, several studies have highlighted the relevance of ferroptosis-related pathways in lymphomas, CLL, and MM, particularly in the context of disease progression metabolic adaptation and resistance to conventional therapies.

### 4.1. Dysregulation of Iron Metabolism

Alterations in iron metabolism are increasingly recognized as a hallmark of lymphoid malignancies and represent a central determinant of ferroptotic sensitivity. Malignant lymphoid cells frequently exhibit enhanced iron uptake and accumulation, reflecting the elevated metabolic demands associated with rapid proliferation and sustained mitochondrial activity. Increased expression of transferrin receptors and dysregulated intracellular iron trafficking contribute to expansion of the labile iron pool, thereby promoting the generation of reactive oxygen species through iron-dependent redox reactions [12].

At the same time, perturbations in ferritin dynamics further influence iron homeostasis in these malignancies. Ferritin serves as a major intracellular iron storage complex, limiting the availability of redox-active iron and protecting cells from oxidative damage. However, enhanced ferritin turnover, particularly through ferritinophagy, can release substantial amounts of ferrous iron into the cytosol, amplifying oxidative stress and sensitizing cells to ferroptosis. Conversely, increased ferritin expression may function as a protective mechanism by buffering intracellular iron and restricting lipid peroxidation. This dual role highlights the complexity of iron regulation in lymphoid tumors and underscores its context-dependent effects on ferroptotic susceptibility [13].

Iron overload has been described in several hematologic malignancies, particularly in heavily transfused patients and in those with altered systemic iron regulation. Excess intracellular iron not only sustains proliferation but also increases vulnerability to ferroptotic death by facilitating lipid peroxide formation. Consequently, malignant lymphoid cells often exist near the threshold of oxidative toxicity, relying on adaptive antioxidant responses to avoid lethal accumulation of lipid peroxides. This condition suggests that therapeutic strategies aimed at further increasing intracellular iron availability or disrupting iron storage mechanisms may effectively enhance ferroptosis induction in selected tumors, although potential systemic toxicity must be carefully considered.

### 4.2. Oxidative Stress and Redox Vulnerability

LPDs are characterized by persistently elevated oxidative stress resulting from aberrant metabolism, mitochondrial dysfunction, oncogenic signaling, and dysregulated iron handling. Increased production of ROS contributes to genomic instability, and disease progression, while simultaneously imposing substantial oxidative pressure on malignant cells. In diffuse large B-cell lymphoma (DLBCL), CLL, and MM, elevated ROS levels have been consistently associated with enhanced proliferation, resistance to apoptosis, and adaptation to microenvironmental stress [14].

Despite this pro-oxidant state, malignant lymphoid cells remain highly dependent on antioxidant systems to preserve redox homeostasis and prevent excessive lipid peroxidation. Activation of transcriptional programs involved in oxidative stress responses, particularly those regulated by NRF2, promotes the expression of detoxifying enzymes and antioxidant proteins that counterbalance ROS accumulation. Enhanced glutathione synthesis, increased cystine uptake, and reinforcement of lipid peroxide detoxification pathways collectively allow tumor cells to tolerate otherwise cytotoxic oxidative conditions [15].

This adaptive antioxidant response, although essential for survival, also creates a state of redox vulnerability or “redox addiction”. Because malignant cells operate under chronically elevated oxidative stress, even modest disruption of antioxidant defenses can rapidly shift the balance toward uncontrolled lipid peroxidation and ferroptotic death. This phenomenon is particularly relevant in therapy-resistant disease, where dependence on redox buffering systems becomes even more pronounced. Consequently, targeting oxidative stress adaptation has emerged as a promising strategy to selectively induce ferroptosis in lymphoid malignancies.

### 4.3. Role of SLC7A11 and GPX4

Among the molecular regulators of ferroptosis, SLC7A11 and GPX4 occupy central positions in determining ferroptotic resistance in LPDs. Increased expression of SLC7A11 has been documented in several lymphoid malignancies and is closely associated with enhanced antioxidant capacity. By promoting cystine uptake and sustaining intracellular glutathione synthesis, SLC7A11 enables malignant cells to maintain lipid redox homeostasis despite elevated oxidative stress. This adaptation provides a major survival advantage and contributes to resistance against both chemotherapy and targeted therapies [16].

Similarly, GPX4 represents a critical defense mechanism against ferroptosis through its ability to detoxify lipid hydroperoxides before they reach cytotoxic levels. Dependence on GPX4 appears particularly pronounced in malignant cells characterized by high oxidative metabolism and abundant polyunsaturated membrane lipids. Experimental inhibition of GPX4 has been shown to induce extensive lipid peroxidation and ferroptotic cell death in lymphoma and myeloma models, highlighting its relevance as a potential therapeutic target [17].

The clinical significance of these pathways is increasingly evident. Elevated expression of SLC7A11 and GPX4 has been associated with aggressive disease behavior, therapeutic resistance, and poor prognosis in multiple hematologic malignancies. Their upregulation may therefore serve not only as a biomarker of ferroptosis resistance but also as an indicator of metabolic adaptation and redox dependency. These observations support the rationale for pharmacological strategies aimed at inhibiting cystine transport or impairing GPX4 activity in order to overcome treatment resistance and enhance tumor cell susceptibility to ferroptosis, particularly in metabolically adapted, relapsed or refractory disease.

### 4.4. Lipid Metabolic Remodeling

Profound alterations in lipid metabolism represent another key feature of LPDs and critically influence ferroptotic sensitivity. Malignant lymphoid cells frequently display changes in membrane composition characterized by increased incorporation of polyunsaturated fatty acids into phospholipids. Because these lipids are highly susceptible to oxidative attack, their enrichment creates a favorable substrate environment for lipid peroxidation and ferroptosis [18].

The regulation of phospholipid remodeling is strongly influenced by enzymes involved in fatty acid activation and membrane incorporation. Among these, acyl-CoA synthetase long-chain family member 4 plays a particularly important role by promoting the esterification of polyunsaturated fatty acids into membrane phospholipids. Increased ACSL4 activity has been closely associated with ferroptotic susceptibility in several cancer models, including hematologic malignancies, where it facilitates the accumulation of oxidizable lipid species. In parallel, lipid oxygenation pathways involving lipoxygenases contribute to the propagation of lipid peroxide formation and amplification of ferroptotic signaling [19].

In addition to enzymatic lipid oxidation, iron-dependent non-enzymatic peroxidation reactions further accelerate membrane damage under conditions of oxidative stress. The interplay between altered membrane composition, elevated reactive oxygen species, and impaired antioxidant defenses ultimately determines the extent of lipid peroxide accumulation and the likelihood of ferroptotic cell death [20].

## 5. Crosstalk with Other Cellular Pathways

Ferroptosis does not occur as an isolated biological process but rather emerges from a complex network of interactions involving multiple cellular pathways that regulate survival, stress adaptation, and cell death. In lymphoproliferative malignancies, the interplay between ferroptosis and other signaling mechanisms is particularly relevant, as malignant lymphoid cells exhibit extensive metabolic plasticity and dynamic responses to oxidative stress. Pathways involved in apoptosis, autophagy, transcriptional regulation, and mitochondrial metabolism collectively influence ferroptotic susceptibility and contribute to the balance between tumor cell survival and death. Understanding these interconnected mechanisms is therefore essential for identifying therapeutic strategies capable of overcoming resistance and enhancing ferroptosis induction.

### 5.1. Interaction with Apoptosis and Autophagy

Although ferroptosis is mechanistically distinct from apoptosis, increasing evidence indicates substantial functional overlap between these forms of regulated cell death. Apoptosis is primarily characterized by caspase activation, chromatin condensation, and membrane blebbing, whereas ferroptosis is driven by iron-dependent lipid peroxidation and catastrophic membrane damage. Nevertheless, both pathways may coexist within the same cellular context and can influence each other through shared upstream stress signals, including mitochondrial dysfunction, ROS accumulation, and metabolic imbalance [21].

In LPDs, resistance to apoptosis represents a major hallmark of disease progression and therapeutic failure. Overexpression of anti-apoptotic proteins, such as members of the BCL-2 family, allows malignant cells to evade conventional cytotoxic therapies. Under these conditions, ferroptosis may serve as an alternative mechanism of cell death capable of bypassing apoptotic resistance. Indeed, experimental studies have shown that tumor cells resistant to apoptosis often retain susceptibility to ferroptosis, particularly when antioxidant defenses are disrupted or lipid peroxidation is enhanced [22].

Autophagy also plays a dual and highly context-dependent role in ferroptosis regulation. While basal autophagy may protect cells from oxidative stress by removing damaged organelles and oxidized macromolecules, selective forms of autophagy can actively promote ferroptotic cell death. Among these, ferritinophagy has emerged as a key regulatory mechanism linking iron metabolism to ferroptosis. Through autophagic degradation of ferritin mediated by nuclear receptor coactivator 4 (NCAO4), intracellular iron stores are mobilized, increasing the labile iron pool and facilitating lipid peroxide formation. Enhanced ferritinophagy therefore amplifies oxidative stress and sensitizes cells to ferroptosis. In LPDs, dysregulation of autophagic pathways may significantly influence ferroptotic vulnerability, particularly in metabolically active and therapy-resistant tumor populations [21].

### 5.2. Transcriptional and Post-Transcriptional Regulation

The susceptibility of malignant lymphoid cells to ferroptosis is strongly influenced by transcriptional and post-transcriptional regulatory networks that coordinate oxidative stress responses, metabolic adaptation, and iron homeostasis. Among the most relevant regulators is p53, which exerts complex and context-dependent effects on ferroptosis. In addition to its established role in apoptosis and cell-cycle arrest, p53 can promote ferroptosis by suppressing SLC7A11 expression, thereby reducing cystine uptake and impairing glutathione synthesis. This effect enhances lipid peroxidation and increases sensitivity to oxidative stress. However, p53 may also exert protective functions under certain conditions by modulating antioxidant pathways and limiting excessive ferroptotic damage, highlighting the multifaceted nature of its regulatory activity.

Another central mediator of ferroptosis resistance is NRF2, a master transcriptional regulator of antioxidant defense. Activation of NRF2 induces the expression of multiple cytoprotective genes involved in glutathione synthesis, iron sequestration, detoxification of reactive oxygen species, and lipid peroxide reduction. In lymphoproliferative disorders, persistent NRF2 activation contributes to adaptation against chronic oxidative stress and promotes survival under metabolically challenging conditions. Increased NRF2 signaling has also been associated with therapeutic resistance, as enhanced antioxidant capacity allows malignant cells to evade ferroptotic death despite elevated oxidative pressure [23].

The oncogenic transcription factor MYC further contributes to ferroptosis regulation through its profound effects on cellular metabolism. MYC-driven tumors display increased metabolic demands and elevated reactive oxygen species production, resulting in heightened dependence on antioxidant systems. At the same time, MYC promotes glutamine utilization, mitochondrial activity, and lipid biosynthesis, all of which influence ferroptotic sensitivity. In aggressive lymphoid malignancies characterized by MYC overexpression, this metabolic rewiring creates a paradoxical state in which enhanced proliferation is accompanied by increased vulnerability to oxidative stress and ferroptosis induction [24].

### 5.3. Metabolic Pathways

Several metabolic pathways critically contribute to the regulation of ferroptosis in lymphoproliferative disorders by influencing redox homeostasis, lipid synthesis, and mitochondrial function. Among these, glutaminolysis plays a central role in sustaining the metabolic demands of malignant lymphoid cells. Enhanced glutamine utilization provides intermediates for the tricarboxylic acid cycle, supports nucleotide and amino acid biosynthesis, and contributes to antioxidant defense through glutathione production. At the same time, increased glutaminolysis promotes mitochondrial oxidative metabolism and reactive oxygen species generation, thereby facilitating conditions favorable for ferroptosis. Inhibition of glutamine metabolism has been shown to modulate ferroptotic sensitivity, underscoring the close relationship between nutrient utilization and lipid peroxidation [25].

The mevalonate pathway also exerts an important influence on ferroptosis regulation through its involvement in cholesterol biosynthesis, membrane organization, and antioxidant defense. This pathway contributes to the synthesis of coenzyme Q10, which functions as a potent lipophilic antioxidant capable of suppressing lipid peroxidation. In addition, mevalonate metabolism supports the maturation of selenoproteins, including GPX4, thereby reinforcing ferroptosis resistance. Dysregulation of this pathway in malignant lymphoid cells may therefore affect both membrane composition and antioxidant capacity, influencing susceptibility to ferroptotic death [26].

Mitochondrial metabolism represents another major determinant of ferroptotic sensitivity. Beyond their role in energy production, mitochondria are important sources of reactive oxygen species and regulators of cellular iron utilization. Altered mitochondrial function in lymphoid malignancies contributes to oxidative stress, metabolic plasticity, and adaptation to nutrient deprivation. Mitochondrial dysfunction may enhance lipid peroxide accumulation through increased reactive oxygen species production and impaired redox buffering, thereby promoting ferroptosis. Conversely, metabolic adaptations that preserve mitochondrial integrity can protect malignant cells from oxidative damage and ferroptotic cell death.

**Figure 1:**
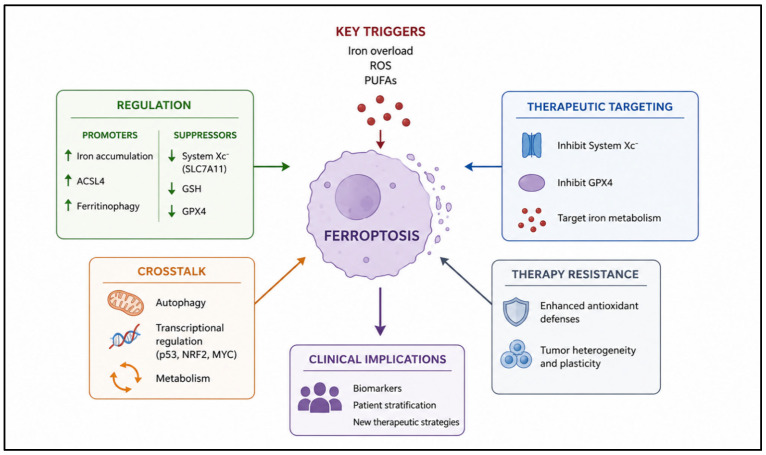
Overview of ferroptosis in Lymphoproliferative Disorders. ACSL4: Acyl-CoA Synthetase Long-Chain Family Member 4; SC7A11: Solute Carrier Family 7 Member 11; GSH: glutathione; GPX4: Glutathione peroxidase 4; NRF2: Nuclear factor erythroid 2-related factor 2; ROS: Reactive oxygen species; PUFAs: Polyunsaturated Fatty Acids. Created with the support of ChatGPT (GPT-5.3).

All pivotal roles and mechanisms of ferroptosis in LPDs are summarized schematically in Figure 1.

## 6. Ferroptosis and Therapy Resistance

Therapeutic resistance remains one of the principal challenges in the management of lymphoproliferative malignancies and is frequently associated with profound metabolic and redox adaptations that allow tumor cells to survive under treatment-induced stress. Scientific evidence suggests that ferroptosis is closely interconnected with mechanisms of resistance to chemotherapy, targeted therapies, and immunotherapy. Malignant lymphoid cells often develop strategies to evade oxidative damage and maintain lipid redox homeostasis, thereby limiting ferroptotic cell death despite exposure to cytotoxic agents. Understanding these adaptive processes is therefore critical for the development of more effective therapeutic approaches capable of overcoming resistance.

Resistance to conventional chemotherapy is frequently associated with enhanced antioxidant defenses and altered iron metabolism. Many chemotherapeutic agents exert part of their antitumor activity through the induction of oxidative stress and reactive oxygen species accumulation. In response, malignant lymphoid cells activate compensatory pathways that increase glutathione synthesis, reinforce lipid peroxide detoxification, and reduce intracellular oxidative damage. Upregulation of antioxidant systems involving SLC7A11, GPX4, and NRF2 enables tumor cells to tolerate persistent oxidative stress and avoid ferroptotic death. In parallel, changes in iron storage and ferritin metabolism may limit the availability of redox-active iron, further reducing susceptibility to lipid peroxidation. These adaptations contribute not only to resistance against cytotoxic chemotherapy but also to the survival of residual tumor populations capable of driving disease relapse [17].

The relationship between ferroptosis and resistance to targeted therapies has also attracted increasing attention. In chronic lymphocytic leukemia and several lymphoma subtypes, targeted inhibitors directed against signaling pathways such as B-cell receptor signaling and anti-apoptotic proteins have substantially improved clinical outcomes. However, resistance inevitably develops in a significant proportion of patients. Emerging evidence indicates that malignant cells escaping targeted therapies frequently undergo metabolic rewiring characterized by increased dependence on antioxidant pathways and altered lipid metabolism. Enhanced cystine uptake, increased glutathione availability, and activation of ferroptosis-suppressive mechanisms allow neoplastic cells to adapt to therapy-induced oxidative stress. In some cases, resistant cells exhibit increased expression of GPX4 and reduced accumulation of lipid peroxides, thereby establishing a ferroptosis-resistant phenotype. These observations suggest that pharmacological induction of ferroptosis may restore therapeutic sensitivity in resistant disease.

Ferroptosis has also emerged as a potential determinant of resistance to immunotherapy. The interaction between tumor cells and the immune microenvironment profoundly influences oxidative stress and metabolic homeostasis within lymphoid malignancies. Activated immune cells, particularly cytotoxic T lymphocytes, can promote lipid peroxidation and ferroptotic signaling in tumor cells through the release of inflammatory cytokines and modulation of cystine metabolism. However, malignant cells may counteract these effects by reinforcing antioxidant defenses and altering iron handling pathways. Chronic exposure to inflammatory stimuli within the tumor microenvironment may further select for cell populations with enhanced resistance to oxidative stress and ferroptosis. In addition, metabolic competition between immune cells and tumor cells for nutrients such as cystine and glutamine may impair effective antitumor immune responses while simultaneously promoting ferroptosis resistance in malignant cells. Although the precise role of ferroptosis in immunotherapy resistance remains incompletely understood, accumulating evidence suggests that modulation of ferroptotic pathways could enhance immune-mediated tumor eradication and improve the efficacy of immune checkpoint inhibitors and cellular therapies [24].

An additional factor contributing to ferroptosis resistance is the remarkable plasticity of malignant lymphoid cells. Tumor populations can dynamically adapt to environmental and therapeutic pressures through extensive metabolic reprogramming, allowing survival under conditions that would otherwise induce oxidative damage and cell death. These adaptive responses include alterations in mitochondrial metabolism, remodeling of membrane lipid composition, activation of stress-response pathways, and modulation of iron homeostasis. By reshaping their metabolic landscape, malignant cells can reduce lipid peroxide accumulation and maintain redox equilibrium despite persistent oxidative stress. Importantly, therapy-resistant cells often exhibit stem cell-like features and increased metabolic flexibility, characteristics that may further enhance their ability to evade ferroptosis and repopulate disease after treatment.

## 7. Therapeutic Targeting of Ferroptosis

The growing recognition of ferroptosis as a critical vulnerability in lymphoproliferative malignancies has stimulated intense interest in the development of therapeutic strategies aimed at selectively inducing this form of regulated cell death. Because malignant lymphoid cells frequently operate under conditions of elevated oxidative stress and exhibit substantial dependence on antioxidant defenses, pharmacological disruption of ferroptosis-regulating pathways may shift the intracellular balance toward lethal lipid peroxidation. Several therapeutic approaches have therefore been investigated, including inhibition of cystine uptake, direct targeting of lipid peroxide detoxification systems, modulation of iron metabolism, and combination strategies designed to enhance treatment efficacy while overcoming resistance mechanisms [12].

### 7.1. Inhibition of System Xc^−^

Among the most extensively studied ferroptosis-inducing strategies is the inhibition of the cystine/glutamate antiporter system Xc^−^, which plays a central role in maintaining intracellular glutathione levels and protecting cells from oxidative stress. By limiting cystine uptake, inhibition of this transporter reduces the availability of cysteine required for glutathione synthesis, thereby impairing antioxidant defenses and promoting lipid peroxide accumulation [27].

Erastin was the first small molecule identified as a ferroptosis inducer acting through inhibition of system Xc^−^. Experimental studies have demonstrated that erastin effectively depletes intracellular glutathione and triggers iron-dependent lipid peroxidation in multiple cancer models, including hematologic malignancies. In lymphoid tumors, sensitivity to erastin appears to correlate with elevated oxidative stress and dependence on cystine metabolism. Several erastin derivatives and structurally related compounds have subsequently been developed with improved pharmacologic properties and enhanced stability, further supporting the therapeutic potential of targeting cystine uptake pathways [14].

In addition to directly inducing ferroptosis, inhibition of system Xc^−^ may sensitize malignant lymphoid cells to other forms of therapy by weakening their antioxidant capacity. Because many therapy-resistant tumor populations exhibit increased reliance on glutathione metabolism, disruption of cystine transport represents a particularly attractive strategy for overcoming adaptive resistance mechanisms, especially when combined with agents that increase ROS production or interfere with mitochondrial function.

### 7.2. GPX4 Inhibitors

Direct inhibition of GPX4 constitutes another major therapeutic strategy for ferroptosis induction. As the principal enzyme responsible for detoxifying lipid hydroperoxides, GPX4 is essential for protecting cellular membranes from oxidative damage. Malignant lymphoid cells characterized by elevated reactive oxygen species production and abundant polyunsaturated membrane lipids appear especially dependent on GPX4 activity for survival [28].

RSL3 is among the best-characterized GPX4 inhibitors and acts by directly inactivating the catalytic function of the enzyme, leading to rapid accumulation of lipid peroxides and ferroptotic cell death. Experimental evidence has demonstrated that RSL3 and related compounds effectively induce ferroptosis in lymphoma and multiple myeloma models, particularly in cells displaying high oxidative stress and increased dependence on glutathione metabolism. Importantly, GPX4 inhibition may remain effective even in tumor populations resistant to apoptosis, supporting its potential role in refractory disease [29].

Several additional compounds targeting GPX4 or related lipid peroxide detoxification pathways are currently under investigation. However, because GPX4 is also essential for the survival of normal tissues, concerns regarding systemic toxicity and therapeutic selectivity remain important challenges for clinical translation. Strategies aimed at selectively targeting malignant cells while minimizing damage to normal tissues will therefore be critical for the future development of GPX4-based therapies.

### 7.3. Targeting Iron Metabolism

Given the fundamental role of iron in ferroptosis execution, modulation of iron metabolism has emerged as another promising therapeutic approach in lymphoproliferative malignancies. Increasing intracellular iron availability can amplify reactive oxygen species generation and promote lipid peroxidation, thereby sensitizing malignant cells to ferroptosis.

Several iron modulation strategies have been explored, including enhancement of intracellular iron accumulation, induction of ferritin degradation, and manipulation of iron transport pathways. Promotion of ferritinophagy increases the release of ferrous iron into the cytosol, expanding the labile iron pool and facilitating oxidative membrane damage. Similarly, upregulation of transferrin receptor expression or inhibition of iron export mechanisms may further augment ferroptotic susceptibility [30].

Conversely, iron chelators have also been investigated in hematologic malignancies, particularly in the context of iron overload associated with repeated transfusions. Although iron chelation can reduce oxidative stress and limit ferroptosis, it may also impair tumor growth by restricting iron availability for proliferation. These apparently opposing effects underscore the complex relationship between iron metabolism and tumor biology, suggesting that therapeutic modulation of iron pathways must be carefully tailored according to disease context, systemic iron status, and metabolic state, and considered in light of potential interactions with ferroptosis-targeting strategies.

### 7.4. Combination Therapies

The integration of ferroptosis induction with established therapeutic modalities represents a particularly promising strategy for enhancing antitumor efficacy and overcoming resistance. Conventional chemotherapeutic agents frequently induce oxidative stress as part of their cytotoxic activity, and combining these agents with ferroptosis inducers may amplify lipid peroxide accumulation beyond the threshold compatible with tumor cell survival [12].

Preclinical studies have demonstrated synergistic effects between ferroptosis induction and chemotherapy in several lymphoid malignancies. Inhibition of antioxidant pathways may sensitize malignant cells to DNA-damaging agents and enhance treatment-induced oxidative injury. This approach appears especially relevant in therapy-resistant tumors, where increased dependence on redox buffering systems creates exploitable vulnerabilities.

Synergistic interactions have also been described between ferroptosis induction and immunotherapy. Cytotoxic T lymphocytes can promote ferroptotic signaling through inflammatory cytokines and modulation of cystine metabolism within the tumor microenvironment. Enhancing ferroptosis may therefore strengthen immune-mediated tumor cell killing and improve the efficacy of immune-based therapies. At the same time, ferroptosis-related changes in tumor metabolism and lipid composition may influence immune cell function, highlighting the complex bidirectional relationship between ferroptosis and antitumor immunity [6].

The combination of ferroptosis-targeting agents with existing therapeutic regimens may therefore represent an effective strategy to eliminate resistant tumor populations while reducing the likelihood of adaptive escape mechanisms, provided that schedule, dosing, and sequencing are optimized and potential additive toxicities are carefully monitored.

Beyond chemotherapy and immunotherapy, ferroptosis induction has shown synergistic interactions with radiotherapy, targeted therapies, and epigenetic modulators in several preclinical models. Such combinations may enhance oxidative stress, overcome adaptive resistance mechanisms, and increase tumor susceptibility to lipid peroxidation, supporting the rationale for multimodal therapeutic approaches.

### 7.5. Preclinical and Clinical Evidence

A growing body of evidence indicates that ferroptosis represents not only a relevant biological mechanism in lymphoid malignancies, but also a clinically actionable metabolic vulnerability, particularly in relapsed/refractory disease characterized by profound oxidative stress adaptation, mitochondrial rewiring, and therapy-induced metabolic selection pressure. Increasing translational data suggest that distinct lymphoproliferative disorders display disease-specific ferroptotic dependencies, involving differential regulation of iron metabolism, glutathione homeostasis, lipid remodeling, and antioxidant signaling pathways [31,32,33].

In DLBCL, ferroptosis has emerged as a particularly relevant mechanism in biologically aggressive molecular subgroups. DLBCL cells frequently exhibit enhanced oxidative phosphorylation, elevated ROS production, increased intracellular iron accumulation, and dependence on the NRF2/SLC7A11/GPX4 antioxidant axis. These alterations are especially pronounced in MYC-driven tumors and in double-hit or double-expressor lymphomas, where metabolic hyperactivation creates a state of chronic oxidative stress close to the threshold of lethal lipid peroxidation. Experimental studies demonstrated that pharmacologic inhibition of GPX4 induces rapid accumulation of oxidized phospholipids and ferroptotic death in DLBCL models, including chemotherapy-resistant clones. Importantly, PRMT5 inhibition has recently been shown to sensitize B-cell lymphoma cells to ferroptosis through impairment of redox adaptation pathways, suggesting a mechanistic interaction between epigenetic regulation and ferroptotic vulnerability. Likewise, inhibition of CISD2/CDGSH iron sulfur domain 2 promotes ferroptosis through suppression of the NRF2/SLC7A11/GPX4 pathway, further supporting the relevance of redox-buffering systems in DLBCL survival [31]. From a clinical perspective, these observations are particularly important because refractory DLBCL is frequently characterized by metabolic adaptation rather than solely genomic resistance. Relapsed disease often exhibits increased antioxidant dependence, mitochondrial plasticity, and enhanced iron sequestration, all of which may constitute exploitable liabilities. TP53-mutated DLBCL appears especially susceptible to ferritinophagy-mediated ferroptosis. APR-246 (eprenetapopt), originally developed as a p53-reactivating compound, has been shown to trigger ferritinophagy, increase the labile iron pool, and induce ferroptotic death in TP53-mutated DLBCL cells. This finding is clinically relevant because TP53 alterations remain among the strongest adverse prognostic factors in aggressive B-cell lymphomas and are associated with resistance to immunochemotherapy and CAR-T-cell therapy. Additional evidence indicates that ferroptosis may contribute to therapeutic responses induced by standard lymphoma treatments. Cytotoxic agents such as anthracyclines and platinum compounds increase ROS production and lipid oxidative damage, potentially priming lymphoma cells toward ferroptosis. Similarly, radiation therapy induces iron-dependent oxidative injury that may synergize with ferroptotic signaling. Consequently, ferroptosis induction may represent a biologically rational strategy to potentiate conventional chemoimmunotherapy in aggressive lymphomas.

In CLL, ferroptosis biology appears closely linked to mitochondrial metabolism and microenvironmental interactions. CLL cells are characterized by increased oxidative phosphorylation, elevated mitochondrial ROS generation, chronic activation of B-cell receptor signaling, and substantial dependence on glutathione metabolism for survival. Unlike highly proliferative lymphomas, CLL cells rely less on glycolysis and more on mitochondrial bioenergetics, rendering them particularly vulnerable to perturbations in redox homeostasis. CLL cells display enhanced expression of antioxidant enzymes and increased cystine dependence mediated by SLC7A11 activity. Stromal interactions within lymph nodes and bone marrow further reinforce antioxidant defenses by providing cysteine and metabolic substrates that support glutathione synthesis. This protective microenvironment may contribute directly to resistance against oxidative stress-mediated cell death. Experimental inhibition of system Xc^−^ or GPX4 has been shown to induce substantial ferroptotic death in primary CLL cells, particularly in metabolically active subsets characterized by unmutated IGHV status and enhanced mitochondrial activity.

The relationship between ferroptosis and targeted therapies in CLL is of particular translational interest. Resistance to BTK inhibitors and venetoclax is increasingly associated with profound metabolic rewiring, including enhanced oxidative phosphorylation, altered fatty acid metabolism, and reinforcement of antioxidant pathways. Venetoclax-resistant CLL cells exhibit increased mitochondrial dependence and elevated ROS buffering capacity, potentially increasing their reliance on GPX4-mediated lipid peroxide detoxification. These observations suggest that ferroptosis induction could represent a therapeutic strategy capable of overcoming apoptosis resistance in heavily pretreated CLL. Moreover, BTK inhibition itself may alter intracellular redox signaling and iron metabolism, potentially sensitizing selected leukemic populations to ferroptotic stress [6].

In MM, ferroptosis has gained major interest because malignant plasma cells exist under constitutively elevated proteotoxic and oxidative stress. Myeloma cells are characterized by intense immunoglobulin synthesis, endoplasmic reticulum stress, increased ROS generation, high iron requirements, and profound dependence on antioxidant systems. Consequently, MM cells operate near the limit of redox tolerance and require continuous activation of glutathione-dependent detoxification pathways to prevent lethal lipid peroxidation. Several studies demonstrated that GPX4 inhibition induces marked ferroptotic death in myeloma models, including high-risk and proteasome inhibitor-resistant disease. Myeloma cells appear highly dependent on GPX4 because proteasome dysfunction, unfolded protein response activation, and mitochondrial stress continuously generate oxidative damage. Inhibition of GPX4 therefore results in catastrophic accumulation of lipid hydroperoxides and membrane destruction. Importantly, ferroptosis induction remains active in bortezomib-resistant populations, suggesting that ferroptosis may bypass canonical apoptosis resistance mechanisms associated with relapsed MM. The interaction between ferroptosis and proteasome inhibition is particularly relevant for clinical hematology. Bortezomib and carfilzomib increase intracellular oxidative stress, impair protein quality control systems, and exacerbate mitochondrial dysfunction. These effects appear to synergize with ferroptosis induction by overwhelming antioxidant buffering capacity. Experimental data suggest that proteasome inhibitors may sensitize myeloma cells to ferroptosis through depletion of intracellular glutathione, increased iron-dependent ROS generation, and accumulation of oxidized membrane lipids. Relapsed/refractory MM often demonstrates increased iron metabolism dysregulation, enhanced ferritin turnover, and altered lipid remodeling, all of which may further amplify ferroptotic susceptibility. Iron metabolism itself may have direct clinical implications in MM. Patients with advanced disease frequently exhibit altered systemic iron homeostasis, transfusion dependence, inflammation-associated iron redistribution, and increased ferritin levels. Although ferritin elevation partly reflects inflammation, intracellular ferritinophagy may paradoxically increase the labile iron pool within plasma cells, promoting ferroptotic priming. Moreover, high-risk molecular subtypes characterized by MYC activation, chromosome 1q amplification, or proteasome inhibitor resistance may exhibit stronger dependence on antioxidant defense pathways, potentially identifying biologically enriched populations for ferroptosis-targeted approaches [32].

Beyond DLBCL, CLL, and MM, additional lymphoma entities have shown evidence of ferroptotic vulnerability. Mantle cell lymphoma (MCL) demonstrates increased oxidative phosphorylation and ROS accumulation, particularly in blastoid variants and BTK inhibitor-resistant disease. Given the strong dependence of MCL on mitochondrial metabolism and BCL2-mediated survival signaling, ferroptosis induction may provide a mechanism to overcome resistance to covalent BTK inhibitors or venetoclax-based combinations.

Burkitt lymphoma represents another potentially ferroptosis-sensitive disease because MYC overexpression profoundly increases metabolic flux, mitochondrial ROS production, glutaminolysis, and lipid biosynthesis. This hypermetabolic phenotype creates substantial oxidative pressure and dependence on glutathione buffering systems. Consequently, MYC-driven lymphomas may be intrinsically predisposed to ferroptotic death when antioxidant pathways are disrupted.

Peripheral T-cell lymphomas and NK/T-cell lymphomas also exhibit profound metabolic dysregulation, inflammatory signaling, and oxidative stress. NK/T-cell lymphoma, in particular, is characterized by high metabolic activity, hypoxia-related signaling, and aggressive tissue destruction associated with chronic inflammatory microenvironments. These biological features may enhance lipid peroxidation and ferroptotic susceptibility, although clinical data remain limited [17].

Another clinically important aspect concerns the interaction between ferroptosis and the immune microenvironment. Activated CD8+ T lymphocytes can promote ferroptosis in tumor cells through IFN-γ-mediated suppression of SLC7A11 expression and disruption of cystine metabolism. This mechanism may partially contribute to the activity of immune checkpoint inhibitors and CAR-T-cell therapy. However, prolonged inflammatory signaling may also select lymphoma clones with reinforced antioxidant defenses and ferroptosis resistance. Understanding the balance between immune-mediated ferroptosis induction and adaptive resistance mechanisms may therefore become highly relevant for future combinatorial strategies integrating immunotherapy and ferroptosis-targeting agents.

Despite rapidly expanding preclinical evidence, clinical translation remains at an early stage. No ferroptosis-targeting therapy has yet been approved in hematologic malignancies, and prospective clinical trials are still limited. Nevertheless, increasing translational data indicate that biomarkers such as GPX4, SLC7A11, ACSL4, NRF2 activation signatures, ferritinophagy markers, mitochondrial ROS levels, and lipid peroxidation products may help identify ferroptosis-sensitive tumors. Future development of ferroptosis-based therapies will likely require biomarker-driven patient selection, rational combination strategies, and disease-specific metabolic profiling to maximize efficacy while limiting systemic toxicity [31,32].

Most currently available evidence regarding ferroptosis in lymphoproliferative malignancies derives from in vitro studies and animal models. Clinical validation remains limited, and no ferroptosis-targeting agents have yet been approved for hematologic malignancies. Nevertheless, several compounds with ferroptosis-modulating properties, including APR-246 (eprenetapopt) and nanoparticle-based formulations, are being investigated in early translational and clinical settings. Future biomarker-driven studies will be necessary to determine the safety and efficacy of ferroptosis-targeting strategies in patients.

Table 2 describes mechanisms, therapeutic implications and translational relevance of ferroptosis in lymphoproliferative disorders.

## 8. Biomarkers of Ferroptosis

The identification of reliable biomarkers of ferroptosis represents a critical step for the clinical translation of ferroptosis-based therapeutic strategies in lymphoproliferative malignancies. Because ferroptosis is regulated by a complex network involving iron metabolism, lipid peroxidation, antioxidant defenses, and metabolic adaptation, multiple molecular and biochemical parameters have been investigated as potential indicators of ferroptotic susceptibility and therapeutic response. The development of robust biomarkers may not only improve patient stratification but also facilitate monitoring of treatment efficacy and identification of resistant tumor populations.

Among the most extensively studied biomarkers are the expression levels of SLC7A11 and GPX4, which play central roles in ferroptosis resistance. Increased SLC7A11 expression reflects enhanced cystine uptake and elevated glutathione synthesis, enabling malignant cells to maintain redox homeostasis under conditions of oxidative stress. Similarly, high GPX4 expression is associated with efficient detoxification of lipid hydroperoxides and protection against ferroptotic membrane damage. In several hematologic malignancies, elevated levels of these proteins have been correlated with aggressive disease behavior, therapy resistance, and reduced sensitivity to oxidative stress-inducing treatments. Their expression patterns may therefore provide valuable information regarding the ferroptotic status of tumor cells and their dependence on antioxidant pathways [33].

Markers of lipid peroxidation have also emerged as important indicators of ferroptosis activation. Since accumulation of oxidized phospholipids is a defining feature of ferroptotic cell death, several lipid-derived products have been investigated as potential biomarkers. Increased levels of malondialdehyde, 4-hydroxynonenal, and oxidized phosphatidylethanolamines have been associated with enhanced lipid oxidative damage and ferroptotic signaling. In addition, accumulation of intracellular lipid reactive oxygen species, detected through fluorescent probes and oxidative stress assays, is frequently used in experimental models to evaluate ferroptosis induction. Although many of these markers remain primarily research tools, they provide important insights into the oxidative state of malignant lymphoid cells and may eventually contribute to clinical assessment of ferroptotic activity.

Beyond individual proteins or oxidative products, increasing attention has been directed toward ferroptosis-related gene signatures. Transcriptomic analyses have identified distinct expression profiles involving genes associated with iron metabolism, lipid remodeling, antioxidant defense, and oxidative stress responses. These signatures may reflect the overall ferroptotic vulnerability of tumor cells and have shown potential prognostic relevance in several malignancies. In lymphoid tumors, specific ferroptosis-associated transcriptional patterns have been linked to disease aggressiveness, metabolic adaptation, and therapeutic resistance. Moreover, integration of ferroptosis-related gene expression with broader molecular classifications may improve risk stratification and provide a more comprehensive understanding of tumor biology [19].

The potential clinical applications of ferroptosis biomarkers are substantial. Assessment of ferroptosis-related pathways may help identify patients most likely to benefit from therapies targeting oxidative stress or lipid peroxide detoxification systems. Biomarkers could also be used to monitor treatment response, detect emerging resistance mechanisms, and guide combination therapeutic strategies involving chemotherapy, targeted agents, or immunotherapy. Furthermore, characterization of ferroptotic profiles may contribute to precision medicine approaches by enabling personalized therapeutic interventions based on the metabolic and redox dependencies of individual tumors. However, before routine clinical implementation, these candidate biomarkers will require analytical validation, harmonization of assays across laboratories, and demonstration of independent prognostic and predictive value in prospective cohorts of patients with lymphoproliferative disorders.

Despite their promising prognostic and predictive potential, ferroptosis-related biomarkers still face several limitations that hinder their routine clinical application. Most candidate biomarkers, including GPX4, SLC7A11, ACSL4 expression and lipid peroxidation products, have been evaluated predominantly in retrospective studies or experimental models. Moreover, the dynamic and context-dependent nature of ferroptosis, interpatient heterogeneity, lack of assay standardization and limited reproducibility among different platforms currently restrict their clinical applicability. Therefore, prospective validation studies and harmonized methodologies will be required before these biomarkers can be incorporated into routine clinical practice.

## 9. Future Perspectives, Current Limitations and Challenges

Despite the growing interest in ferroptosis as a therapeutic vulnerability in LPDs, several important limitations continue to hinder its translation into clinical practice. Although preclinical studies have demonstrated promising antitumor activity through modulation of ferroptotic pathways, the complexity of ferroptosis regulation and its close integration with essential metabolic and redox processes raise significant biological and therapeutic challenges. Addressing these limitations will be essential for the successful development of safe and effective ferroptosis-based treatment strategies [3].

One of the major concerns relates to the specificity of currently available ferroptosis-inducing agents. Many compounds targeting ferroptotic pathways exert pleiotropic effects that extend beyond ferroptosis regulation, complicating the interpretation of experimental findings and potentially limiting therapeutic selectivity. For example, inhibitors of system Xc^−^ and GPX4 may influence multiple aspects of cellular metabolism, oxidative stress signaling, and mitochondrial function in addition to promoting lipid peroxidation. Similarly, several ferroptosis inducers can trigger overlapping forms of regulated cell death, including apoptosis or necroptosis, depending on the cellular context and treatment conditions. These off-target effects create substantial challenges in defining the precise contribution of ferroptosis to antitumor activity and may increase the risk of unintended toxicity in normal tissues [4].

Systemic toxicity represents another major obstacle for the clinical application of ferroptosis-targeting therapies. Because ferroptosis-regulating pathways are also essential for normal cellular homeostasis, pharmacological induction of ferroptosis may damage healthy tissues characterized by high metabolic activity or increased susceptibility to oxidative stress. Organs such as the liver, kidneys, heart, and central nervous system are particularly vulnerable to excessive lipid peroxidation and iron-dependent oxidative injury. Furthermore, prolonged disruption of antioxidant defenses may impair normal immune function and hematopoiesis, raising concerns regarding treatment tolerability in patients with hematologic malignancies who are often heavily pretreated and clinically fragile. The development of tumor-selective delivery systems and more specific ferroptosis modulators will therefore be critical to minimize systemic toxicity while preserving therapeutic efficacy.

Another important challenge is represented by the marked heterogeneity of LPDs. Even within the same diagnostic category, tumor cells may exhibit substantial differences in iron metabolism, antioxidant capacity, lipid composition, and metabolic adaptation, all of which influence ferroptotic sensitivity. In addition, the tumor microenvironment exerts a profound effect on oxidative stress regulation and nutrient availability, further contributing to variability in ferroptotic responses. This heterogeneity complicates the identification of patients most likely to benefit from ferroptosis-based therapies and may facilitate the emergence of resistant cellular subpopulations capable of adapting to oxidative stress. The dynamic metabolic plasticity of malignant lymphoid cells further amplifies this complexity, allowing tumors to rapidly reprogram antioxidant and lipid metabolic pathways in response to therapeutic pressure [12].

The absence of validated and standardized biomarkers also remains a major limitation in the field. Although several candidate biomarkers have been proposed, including expression levels of SLC7A11 and GPX4, lipid peroxidation products, and ferroptosis-related gene signatures, none have yet been fully validated for routine clinical use. The dynamic and context-dependent nature of ferroptosis regulation complicates the interpretation of isolated molecular measurements, while technical variability across experimental platforms limits reproducibility and comparability between studies. Moreover, many currently available biomarkers reflect indirect aspects of oxidative stress rather than ferroptosis-specific processes, reducing their predictive accuracy. The lack of reliable biomarkers not only hampers patient stratification but also limits the ability to monitor treatment response and identify resistance mechanisms during therapy.

Furthermore, future research should also clarify whether ferroptosis represents merely a downstream consequence of oxidative stress or a central driver of clonal evolution and therapeutic resistance in lymphoid malignancies. Longitudinal studies evaluating metabolic adaptation during disease progression and treatment exposure may help identify dynamic ferroptotic states associated with transformation, relapse, or immune escape. In this context, serial assessment of redox metabolism and lipid peroxidation profiles could eventually become integrated into disease monitoring and minimal residual disease evaluation.

Another important future direction involves the investigation of ferroptosis in the context of cellular therapies. Increasing evidence suggests that oxidative stress and lipid metabolic remodeling influence both CAR-T-cell persistence and tumor susceptibility to immune-mediated killing. Understanding how ferroptosis modulation affects the balance between antitumor immunity and immune cell dysfunction may therefore open new opportunities for combination strategies integrating ferroptosis induction with adoptive immunotherapy. In addition, studying ferroptosis in non-malignant hematopoietic and immune compartments will be essential to define tolerability limits and to design regimens that selectively exploit tumor ferroptotic vulnerabilities while preserving or even enhancing antitumor immune responses [34,35,36].

## 10. Conclusions

Ferroptosis is emerging as a biologically relevant and therapeutically exploitable vulnerability in LPDs. Rather than representing an isolated mechanism of regulated cell death, ferroptosis appears deeply integrated with the metabolic architecture of malignant lymphoid cells, linking iron handling, lipid remodeling, mitochondrial activity, and antioxidant dependence. The chronic oxidative stress that characterizes diseases such as DLBCL, CLL, and MM creates a condition of “redox addiction”, in which tumor survival increasingly depends on adaptive pathways centered on SLC7A11, GPX4, NRF2, and glutathione metabolism [37,38].

This metabolic dependency may become even more pronounced during disease progression and therapeutic selection pressure. Relapsed and refractory lymphoid malignancies frequently acquire enhanced antioxidant capacity, altered mitochondrial fitness, and profound metabolic plasticity, all of which contribute to resistance against chemotherapy, targeted agents, and immunotherapy. At the same time, these adaptations may paradoxically increase susceptibility to ferroptosis induction, particularly when lipid peroxide detoxification systems or iron homeostasis are disrupted. In this context, ferroptosis may represent a mechanism capable of bypassing apoptosis resistance, a major hallmark of advanced hematologic malignancies [9,12,39].

Current evidence suggests that ferroptosis-based therapeutic strategies are unlikely to function as standalone approaches, but rather as components of rational combinatorial regimens. Integration with proteasome inhibitors, BTK inhibitors, BCL2 inhibitors, epigenetic therapies, immunotherapy, or conventional chemoimmunotherapy may amplify oxidative stress beyond the threshold compatible with tumor survival. The identification of disease-specific ferroptotic profiles and metabolic signatures will therefore be essential to optimize patient selection and therapeutic efficacy [20,34].

Despite substantial progress in understanding ferroptosis biology, important challenges remain before clinical implementation can be achieved. Tumor heterogeneity, dynamic metabolic adaptation, systemic toxicity, and the lack of validated biomarkers continue to limit translational applicability. Furthermore, the dual role of oxidative stress in both tumor suppression and normal tissue injury underscores the need for selective therapeutic strategies capable of targeting malignant cells while preserving physiological redox homeostasis [18].

Nevertheless, the rapid expansion of translational research in this field strongly supports the clinical relevance of ferroptosis in hematologic oncology. Future studies integrating molecular profiling, metabolomics, and functional redox characterization may help define ferroptosis-sensitive subsets across lymphoid malignancies and facilitate the development of personalized therapeutic approaches. A deeper understanding of the interplay between ferroptosis, the tumor microenvironment, and immune regulation will likely represent a critical step toward the incorporation of ferroptosis-targeting strategies into the therapeutic landscape of modern hematology [2,34].

## Figures and Tables

**Table 1:** Molecular mechanisms of ferroptosis.

Mechanism	Key Players	Function in Ferroptosis
Iron Metabolism	Transferrin, TFRC, DMT1, Ferritin, Ferroportin, LIP	Redox-active iron drives ROS production → lipid peroxidation
Lipid Peroxidation	PUFAs, ACSL4, LPCAT3, LOXs	Oxidative damage to membrane lipids executes ferroptotic death
Antioxidant Defense	GSH, GPX4, System Xc^−^, FSP1, CoQ10, DHODH, GCH1-BH4	Neutralizes lipid peroxides, preventing ferroptosis

**Table 2:** Main mechanisms, therapeutic implications and translational relevance of ferroptosis in lymphoproliferative disorders.

Aspect	Main Mechanisms	Therapeutic Implications
Iron metabolism	Increased LIP, ferritinophagy, ROS generation	Enhanced ferroptotic vulnerability
Oxidative stress	Chronic ROS accumulation, redox addiction	Targeting antioxidant systems
SLC7A11/GPX4	Increased antioxidant defense	Potential therapeutic targets
Lipid remodeling	ACSL4, PUFA enrichment	Increased lipid peroxidation
Therapy resistance	Metabolic adaptation and antioxidant pathways	Ferroptosis induction may restore sensitivity
Combination therapies	Chemotherapy, immunotherapy, radiotherapy	Synergistic antitumor effects
Biomarkers	GPX4, SLC7A11, ACSL4, lipid products	Patient stratification

## Data Availability

No new data were created or analyzed in this study. Data sharing is not applicable to this article.
